# Impact of time to initiation of appropriate antibiotic therapy on early mortality of patients with septic shock

**DOI:** 10.1186/cc12009

**Published:** 2013-03-19

**Authors:** JP Quenot, C Binquet, S Vinault, A Pavon

**Affiliations:** 1 University Hospital Dijon, France; 2 CHU de Dijon, Centre d'Investigation Clinique - Epidémiologie Clinique, Dijon, France

## Introduction

The impact of appropriate antibiotic therapy on prognosis of patients with septic shock is well established. However, the prognostic influence of time to initiation of antibiotics remains debated. We evaluated the effect on 7-day mortality of time to initiation of appropriate antibiotic therapy in patients hospitalised in critical care for septic shock.

## Methods

Secondary analysis from the EPISS cohort. We included only patients admitted to the University Hospital Dijon. Septic shock was defined as initiation of vasopressors in a patient with suspected or documented infection with at least one criterion of hypoperfusion (metabolic acidosis and/or renal insufficiency and/or hepatic dysfunction). We excluded patients with no available bacteriological data. Anti-biotherapy was considered appropriate if at least one of the antibiotics prescribed was active against the germ identified. Bivariate and multivariate logistic regression analysis was used to assess the impact of time to initiation of appropriate antibiotherapy on 7-day mortality.

## Results

In total, 383 patients were admitted with septic shock, of whom 253 (66%) were included; 231(92%) had appropriate antibiotic therapy, of whom 52 (22.5%) died at 7 days. Average time to initiation of appropriate antibiotic therapy was 9 ± 23 hours. By bivariate analysis, body mass index (BMI) <20, SAPS II ≥56, SOFA score ≥11 and bacteremia were significantly associated with 7-day mortality. Urinary tract infection (UTI) was a protective factor. Age, sex, comorbidities (particularly immunosuppression), Knaus score, nosocomial infection and type of germ had no influence on 7-day mortality. By multivariate logistic regression, BMI <20 (OR = 4.87, 95% CI3= 1.36 to 17.43, *P *= 0.01) and SOFA score ≥11 (OR = 7.99, 95% CI = 3.11 to 20.5, *P <*0.001) were the only factors significantly associated with 7-day mortality. UTI was a significant protective factor (OR = 0.30, 95% CI = 0.10 to 0.88, *P *= 0.03). Time to initiation of appropriate antibiotherapy was not associated with 7-day mortality (OR = 0.99, 95% CI = 0.99 to 1.00, *P *= 0.48).

## Conclusion

Prognosis at 7 days of patients with septic shock is largely related to the number of failing organs. The majority received appropriate antibiotic therapy although time to initiation is longer than recommended. Further efforts are warranted to reduce mortality in patients with septic shock.

